# The social determinants of multimorbidity in South Africa

**DOI:** 10.1186/1475-9276-12-63

**Published:** 2013-08-20

**Authors:** Olufunke Alaba, Lumbwe Chola

**Affiliations:** 1 Health Economics Unit, School of Public Health and Family Medicine, University of Cape Town, Cape Town, South Africa; 2 Health Systems and Services Research Unit, Division of Community Health, Stellenbosch University, Cape Town, South Africa

**Keywords:** Multimorbidity, South Africa, Social determinants of health

## Abstract

**Introduction:**

Multimorbidity is a growing concern worldwide, with approximately 1 in 4 adults affected. Most of the evidence on multimorbidity, its prevalence and effects, comes from high income countries. Not much is known about multimorbidity in low income countries, particularly in sub-Saharan Africa. The aim of this study was to determine the prevalence of multimorbidity and examine its association with various social determinants of health in South Africa.

**Method:**

The data used in this study are taken from the South Africa National Income Dynamic Survey (SA-NIDS) of 2008. Multimorbidity was defined as the coexistence of two or more chronic diseases in an individual. Multinomial logistic regression models were constructed to analyse the relationship between multimorbidity and several indicators including socioeconomic status, area of residence and obesity.

**Results:**

The prevalence of multimorbidity in South Africa was 4% in the adult population. Over 70% of adults with multimorbidity were females. Factors associated with multimorbidity were social assistance (Odds ratio (OR) 2.35; Confidence Interval (CI) 1.59-3.49), residence (0.65; 0.46-0.93), smoking (0.61; 0.38-0.96); obesity (2.33; 1.60-3.39), depression (1.07; 1.02-1.11) and health facility visits (5.14; 3.75-7.05). Additionally, income was strongly positively associated with multimorbidity. The findings are similar to observations made in studies conducted in developed countries.

**Conclusion:**

The findings point to a potential difference in the factors associated with single chronic disease and multimorbidity. Income was consistently significantly associated with multimorbidity, but not single chronic diseases. This should be investigated further in future research on the factors affecting multimorbidity.

## Background

Multimorbidity can be defined as the simultaneous occurrence of two or more chronic health conditions in the same person, without defining a primary disease [1-3]. Often, the study of multimorbidity is associated with aging [3]. However, there has been an increase in the prevalence of multimorbidity among people below the age of 65 years [4]. It is estimated that in developed countries, about 1 in 4 adults experience multimorbidity, with half of older adults having 3 or more chronic conditions [3,5]. Multimorbidity impacts negatively on individuals and the health system as a whole. It is associated with increased mortality, poor quality of life and an increased demand on healthcare utilisation, all of which place a strain on healthcare resources [6,7]. Persons with multimorbidity may also be at risk of sub-optimal care due to the high likelihood of obtaining conflicting advice on treatment and care [8,9]. It is often difficult to obtain meaningful medical advice on combinations of chronic conditions faced by an individual due to the pure single-disease outlook prominent in most health systems.

The burden of multimorbidity has typically been studied in high income countries, where its prevalence and socio-economic determinants have been established [2] as well as its impact on healthcare utilization [10], with very few of these studies conducted in low and middle income countries [11-13]. As such, the prevalence and determinants of multimorbidity in sub-Saharan Africa is relatively unknown. This is despite the fact that obesity, a primary predisposing risk factor underlying chronic diseases, is on the increase [14], in developing countries as well as several other chronic non-communicable diseases [15,16]. The increased burden of non-communicable diseases in sub-Saharan Africa has potential implications on the health system and resource allocation [17]. Managing multimorbidity is thus very important. However, this becomes increasingly difficult to do with the absence of adequate information to influence priority setting and decision making. There is thus need to understand the root causes of disease and to provide evidence of the factors affecting both single chronic diseases and multimorbidity [18].

This study seeks to determine the prevalence of multimorbidity in South Africa, and to examine its association with various social determinants of health. In addition to age and gender, structural and intermediary determinants such as socioeconomic status, psychosocial, and environmental factors that affect health are assessed. The WHO social determinants of health framework is used to guide the analysis.

### Theoretical framework

Many researchers have analysed the social determinants of health, showing how factors such as marital status, socioeconomic status, education and smoking influence chronic diseases [14,19], however studies on the social and economic factors associated with multimorbidity are limited [4,20,21]. In this paper, we use the Commission on Social Determinants of Health (CSDH) model to examine the determinants of multimorbidity [22]. The model posits that besides inherent genetic factors, other factors including the living and working environment, life events, behavioural risk factors and socioeconomic status may affect the occurrence and intensity of disease. The model uses structural and intermediary determinants to explain factors influencing health and wellbeing.

Structural determinants include factors related to socioeconomic status, such as education, income and occupation and the broader social opportunity structures, such as social class and gender, which determine access to health care [4]. Generally, economically deprived individuals are more likely to experience the worst health outcomes [23]. A recent study showed that socioeconomic deprivation is associated with an increased likelihood of multimorbidity. Addressing the structural determinants of health is therefore important to creating an enabling environment for equitable delivery of healthcare.

Intermediary determinants of health include material circumstances of living and working conditions and food availability, biological and psychosocial factors. Understanding intermediary factors is particularly important for improving health systems, because these factors address access to health and social participation, which differ by age, gender and ethnicity. An ecological study by Kwachi, et al. found social allegiances and poor relational trust to be linked to mortality [24]. Social capital serves as an important asset that helps to strengthen health information dissemination and improves access to healthcare [25].

## Methods

### Data

Data were taken from the first wave of the 2008 South African National Income Dynamics Study (SA-NIDS). This cross-sectional study was undertaken by the South African Labour and Development Research Unit (SALDRU) based at the University of Cape Town (UCT). A stratified, two-stage cluster sample design was employed in sampling the households to be included in the study. The explicit strata in the Master Sample were the 53 district councils (DCs) in the country. The sample was proportionally allocated to the strata based on the Master Sample DC PSU allocation. A total of 400 Primary Sampling Units (PSUs) were randomly selected within the strata. Within each PSU, 8 non-overlapping samples (clusters) of dwelling units were systematically drawn. The SA-NIDS provides baseline data on a sample of 28,247 individuals including children from 7,301 households, out of a possible 10,642 households, a response rate of 69%. The sample consisted of 16,800 adults from age 15 years. Study approval was granted by the Ethics Committee of the University of Cape Town. A detailed report on the SA-NIDS methodology is provided elsewhere [26]. Household and adult questionnaires were administered to all household members aged 15 years and older. The study documents the dynamic structure of household members and changes in their incomes, expenditures, assets, access to services, education, health and other dimensions of well-being. For this analysis, we applied a cut off age of 18 years, given that multimorbidity was not observed among respondents below 18 years. Thus, observations under the age of 18 years were dropped. Missing data on social and economic indicators, body mass index, age, sex and race and multimorbidity were deleted. The final sample used in the analysis was therefore n = 11,638.

### Measures

#### Dependent variable

In the SA-NIDS, respondents were asked to state whether or not they currently had one or more of several chronic health conditions including tuberculosis (TB), high blood pressure, diabetes or high blood sugar, stroke, asthma and cancer as confirmed by a doctor. In the analysis, these health conditions were combined to create a summative index of multi-morbidity ranging from 0 to 4. A categorical variable was then created to indicate: 0) no chronic disease, 1) presence of 1 chronic disease and 2) multimorbidity (i.e. presence of 2 or more chronic diseases).

#### Independent variables

##### Structural determinants

Based on the CSDH framework, age, gender, race, education, employment, income, social assistance and residence were structural determinants of health. We hypothesized that structural determinants were directly or indirectly associated with multimorbidity. We expected age, gender, race, income and social assistance to be positively associated with multimorbidity. In the literature, multimorbidity is shown to increase with age and females usually have higher multimorbidity prevalence than males [27]. We also expected income and race to be positively associated with multimorbidity. It has been shown that persons from affluent communities and the well to do in most African countries are more likely to experience chronic diseases [19]. However, living in a rural area, employment and education have been shown to be positively related to self-reported health [28,29], thus we expected these variables to be negatively associated with multimorbidity. Social assistance on the other hand was expected to be positively associated with multimorbidity, since the recipients, who are expected to be extremely poor, may be prone to non-communicable diseases such as mental distress and obesity, which are predisposing factors for chronic disease [19].

Age was measured in single years from 18 years and included as a continuous variable. Gender was categorized as 1 – female and 0 – male. Race was a categorical variable with 1 – African, 2 – Coloured, 3 – Asian/Indian and 4 – White. Education was measured in years of schooling and categorised as 1 – no education, 2 – primary (1–7 years), 3 – secondary (8–12 years) and 4 – tertiary (13+ years). Employment included those in both formal and informal employment and was dichotomised into (1) employed and (0) unemployed. The household income was categorically coded into 5 quintiles, ranging from the poorest to the richest quintile as given in the data set. Social assistance was measured by summing the number of government grants available to individuals. This was categorised into 0) no grant, 1) receipt of grant. We included the rural/urban dichotomy to indicate place of residence with (1) rural and (0) urban.

##### Intermediary determinants

In the CSDH framework, health and psychosocial factors are considered to be intermediary factors. We included risk factors of multimorbidity, smoking and obesity, depression, health facility visits and civic participation as intermediary factors. We anticipated that all the intermediary factors would be positively associated with multimorbidity.

Smoking was included as a dichotomous variable indicating 0) non-smoker and 1) smoker. Obesity was estimated from the weights and heights of respondents in the SA-NIDS survey. To measure obesity, we used the body mass index (BMI) as weight (measured in kilograms, kgs) divided by height squared (metres – m^2^), which were measured by enumerators during data collection. A BMI greater than 30 was considered to be obese. A binary variable was thus generated, 1 if obese and 0 otherwise. We expected a positive relationship with multimorbidity as observed with chronic diseases [30]. The variable ‘Depression’ was created using the self-reported Centre for Epidemiologic Depression (CES-D) 10 item scale, which has been validated and shown to correlate with the 20-item CES-D scale [31]. The CES-D is designed to measure depression in the general population. In the SA-NIDS, respondents were asked to indicate on a scale from 1 (rarely) to 4 (all the time) how they felt in the week prior to the interview with regard to the following: whether the respondent felt unusually bothered by things; had trouble concentrating; felt depressed; felt that doing things was an effort; was hopeful about the future; was fearful; had restless sleep; was happy; felt lonely; or could not get going. The depression score was created as a summative index of the 10 items, ranging from 0 to 30, reflecting an increase in depression with increasing score. We used the Cronbach’s Alpha test to check the reliability of the scale. (This gave a scale reliability coefficient of 0.79). A dichotomous variable was created indicating whether an individual had a health facility contact within the month prior to the survey (1 – yes and 0 – no). This was included to control for healthcare utilization [10] because it is expected that the use of health care facilities increases with high prevalence of multimorbidity. Emerging evidence shows that individual level social capital which defines how people connect with others in their environment is a strong positive social determinant of health [32,33]. In the SA-NIDS, respondents were asked to indicate whether they belonged to one or more of 18 associations. This was used as an indicator of individual level social capital, with a dichotomous variable created to reflect whether (1) an individual belonged to at least one group or (0) did not belong to any group.

### Data analysis

All statistical analyses were done in STATA software version 12 (Stata Corp. Inc. TX, USA). Clustering and survey design effects were accounted for using Stata’s stratified multi-stage design feature. A multinomial logistic regression analysis was used to evaluate the unadjusted association of each variable with 1 chronic disease and 2 or more chronic diseases (multimorbidity); the reference category was no chronic disease. Additionally, a multinomial logistic regression was used to control for potentially confounding effects of other variables, with the analysis done in steps guided by the CSDH framework. The multinomial logistic regression was thus performed in 4 steps, with step 1 being the univariate or unadjusted analysis, step 2 included structural variables only, step 3 included intermediary variables only, and step 4 included all the variables. Effect modification was tested, and since no interaction terms were statistically significant at p = 0.05, they were not included in the analysis.

## Results

The sample consisted of 11,638 adults, of which most were females (61%), Table 1. The majority of the respondents had secondary (54%) and only a few had tertiary (6%) education. The proportion of unemployed was 63%, with over 40% receiving social assistance in the form of government grants. The mean age (Standard Deviation – SD) was 40 years (16.7). The mean (SD) depression score was 8.4 (4.8). The mean (SD) monthly household income was R3,123 (6,413). About 53% of respondents lived in rural areas, 22% were smokers, 26% were obese and 27% had had more than 1 or more hospital visits.

**Table 1:** Descriptive statistics of variables used in the analysis

	**None**	**One**	**Two or more**	**Total**
Chronic diseases	(n = 9,195; 79%)	(n = 1,980; 17%)	(n = 463; 4%)	(n = 11,638)
Gender				
Male	3,853 (42% )	586 (30%)	119 (26%)	4,558 (39%)
Female	5,342 (58%)	1,394 (70%)	344 (74%)	7,080 (61%)
Race				
African	7,528 (82%)	1,463 (74%)	333 (72%)	9,324 (80%)
Coloured	1,121 (12%)	339 (17%)	86 (19%)	1,546 (13%)
Asian/Indian	106 (1%)	26 (1%)	15 (3%)	147 (1%)
White	440 (5 %)	152 (8%)	29 (6%)	621 (5%)
Education				
None	1,142 (12%)	520 (26%)	124 (27%)	1,786 (15%)
Primary	1,986 (22%)	707 (36%)	193 (42%)	2,886 (25%)
Secondary	5,455 (59%)	648 (33%)	124 (27%)	6,227 (54%)
Tertiary	612 (7%)	105 (5%)	22 (5%)	739 (6%)
Employment				
Unemployed	5,614 (61%)	1,391 (70%)	351(76%)	7,356 (63%)
Employed	3,581 (39%)	589 (30%)	112 (24%)	4,282 (37%)
Income (quintiles)				
Quintile 1	1,752 (19%)	292 (15%)	37 (8%)	2,081 (18%)
Quintile 2	1,979 (22%)	426 (22%)	83 (18%)	2,488 (21%)
Quintile 3	2,060 (22%)	467 (24%)	143 (31%)	2,670 (23%)
Quintile 4	2,055 (22%)	505 (26%)	130 (28%)	2,690 (23%)
Quintile 5	1,349 (15%)	290 (15%)	70 (15%)	1,709 (15%)
Social assistance				
No	5,873 (64%)	809 (41%)	131 (28%)	6813 (59%)
Yes	3,324 (36%)	1170 (59%)	331 (72%)	4825 (41%)
Residence				
Urban	4,198 (46%)	999 (50%)	236 (51%)	5,433 (47%)
Rural	4,997 (54%)	981 (50%)	227 (49%)	6,205 (53%)
Smoking				
No	7,098 (77%)	1 560 (79%)	379(82%)	9 036 (78%)
Yes	2,098 (23%)	420 (21%)	84 (18%)	2 602 (22%)
Obesity				
Not obese	7,172 (78%)	1,218 (62%)	238 (51%)	8,628 (74%)
Obese	2,023 (22%)	762 (38%)	225 (49%)	3,010 (26%)
Health facility visits				
No	7,470 (81%)	888 (45%)	150 (32%)	8,508 (73%)
Yes	1,725 (19% )	1,092 (55%)	313 (68%)	3,130 (27%)
Civic participation				
No	5,945 (65% )	1,224 (62%)	250 (54%)	7,419 (64%)
Yes	3,250 (35% )	756 (38%)	213 (46%)	4,219 (36%)

**Figure 1:**
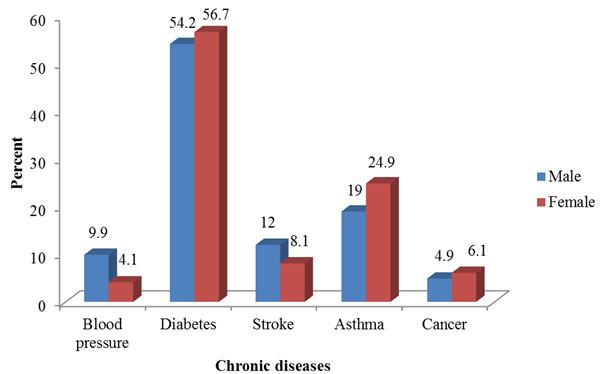
Contribution of chronic diseases to multimorbidity by gender.

Approximately 21% of the respondents had at least 1 chronic disease and 4% had experienced 2 or more chronic diseases i.e. multimorbidity. Among the chronic diseases, diabetes ranked as the highest contributor to multimorbidity with 56%, followed by asthma (23.4%), stroke (9.2%), cancer (5.8%) and blood pressure (5.6%). Figure 1 shows the contribution of chronic diseases to multimorbidity by gender. For both men (54%) and women (57%), diabetes was the highest contributor to multimorbidity. The least contributor among women was blood pressure (4%), and cancer for men (5%).

As shown in Table 1, the majority of respondents with multimorbidity were females (74%). Most of the persons with multimorbidity had less than secondary education (69%), were unemployed (76%), were recipients of at least 1 government grant (78%), and lived in urban areas (51%). About 18% were smokers, 49% were obese and 68% had at least 1 hospital visit in the 30 days prior to the interview.

Table 2 shows the results of the univariate and multivariate multinomial logistic regression of factors associated with single and multimorbidity, relative to the base category no disease. In step 1 (univariate), all variables except smoking and civic participation were significantly associated with 1 chronic disease. The variables not associated with 2 or more chronic diseases (multimorbidity) were residence and civic participation. In comparison to 1 chronic disease, almost all the odds ratios (ORs) of variables associated with multimorbidity were significantly elevated. For example, the OR of health facility visits was 4.94 and 8.05 for 1 chronic disease and multimorbidity, respectively.

**Table 2:** Logistic regression analyses of factors affecting multimorbidity

	**Step 1** - **Unadjusted**		**Step 2** - **Structural**		**Step 3** - **Intermediary**		**Step 4** - **Full**	
	OR	(95% CI)	OR	(95% CI)	OR	(95% CI)	OR	(95% CI)
**1 Chronic disease**								
Age	1.06**	(1.05, 1.07)	1.18**	(1.14, 1.21)			1.15**	(1.11, 1.18)
Gender								
Male	1		1				1	
Female	1.76**	(1.50, 2.06)	1.53**	(1.30, 1.80)			1.18	(0.98, 1.41)
Race								
African			1				1	
Coloured	1.73**	(1.35, 2.20)	1.38*	(1.00, 1.90)			1.33	(0.94, 1.86)
Asian/Indian	1.18	(0.77, 1.82)	1.04	(0.57, 1.89)			0.87	(0.39, 1.95)
White	2.07**	(1.36, 3.17)	1.44	(0.87, 2.40)			1.80*	(1.10, 2.94)
Education								
None			1				1	
Primary	0.85	(0.65, 1.11)	1.24	(0.95, 1.62)			1.30*	(1.01, 1.68)
Secondary	0.30**	(0.24, 0.38)	0.74*	(0.55, 0.99)			0.79	(0.59, 1.05)
Tertiary	0.46*	(0.24, 0.86)	0.73	(0.43, 1.25)			0.83	(0.48, 1.43)
Employment								
Unemployed							1	
Employed	0.68**	(0.58, 0.79)	0.70**	(0.58, 0.86)			0.72**	(0.58, 0.90)
Income								
Quintile 1	1		1				1	
Quintile 2	1.16	(0.93, 1.44)	1.02	(0.81, 1.29)			1.03	(0.81, 1.32)
Quintile 3	1.30*	(1.03, 1.66)	1.20	(0.93, 1.56)			1.21	(0.93, 1.59)
Quintile 4	1.40*	(1.08, 1.83)	1.41*	(1.04, 1.92)			1.44*	(1.05, 1.97)
Quintile 5	1.32	(0.96, 1.83)	1.24	(0.87, 1.77)			1.20	(0.82, 1.75)
Social assistance								
No			1				1	
Yes	2.67**	(2.25, 3.17)	1.48**	(1.22, 1.80)			1.30*	(1.06, 1.60)
Residence								
Urban	1		1				1	
Rural	0.80*	(0.66, 0.97)	0.65**	(0.52, 0.81)			0.70**	(0.58, 0.85)
Smoking								
No	1				1		1	
Yes	0.86	(0.69, 1.08)			1.13	(0.91, 1.41)	0.97	(0.77, 1.22)
Obesity								
Not obese	1				1		1	
Obese	2.82**	(2.37, 3.35)			2.67**	(2.21, 3.23)	1.74**	(1.43, 2.12)
Depression	1.05**	(1.03, 1.07)			1.04**	(1.02, 1.05)	1.03**	(1.01, 1.05)
Health facility								
No	1				1		1	
Yes	4.94**	(4.02, 6.08)			4.56**	(3.74, 5.57)	3.77**	(3.09, 4.60)
Civic participation								
No	1				1		1	
Yes	1.11	(0.94, 1.30)			1.01	(0.85, 1.19)	0.92	(0.77, 1.10)
**2 or more chronic diseases**								
Age	1.08**	(1.07, 1.09)	1.44**	(1.32, 1.57)			1.38**	(1.25, 1.51)
Gender								
Male	1		1				1	
Female	2.44**	(1.77, 3.35)	1.95**	(1.39, 2.73)			1.17	(0.78, 1.75)
Race								
African	1		1				1	
Coloured	1.82**	(1.33, 2.49)	1.24	(0.82, 1.87)			1.43	(0.90, 2.26)
Asian/Indian	2.94*	(1.07, 8.04)	2.13	(0.69, 6.59)			2.12	(0.59, 7.53)
White	2.21*	(1.08, 4.51)	1.12	(0.41, 3.06)			1.92	(0.70, 5.29)
Education								
None	1		1				1	
Primary	0.79	(0.55, 1.13)	1.23	(0.84, 1.80)			1.34	(0.92, 1.97)
Secondary	0.27**	(0.17, 0.42)	0.91	(0.56, 1.48)			0.98	(0.58, 1.66)
Tertiary	0.18**	(0.09, 0.34)	0.30	(0.08, 1.16)			0.40	(0.11, 1.44)
Employment								
Unemployed	1		1				1	
Employed	0.48**	(0.34, 0.70)	0.65*	(0.42, 0.99)			0.70	(0.46, 1.07)
Income								
Quintile 1	1		1				1	
Quintile 2	2.80**	(1.59, 4.96)	2.14*	(1.21, 3.79)			2.23*	(1.28, 3.88)
Quintile 3	3.48**	(1.97, 6.16)	2.90**	(1.63, 5.16)			3.02**	(1.71, 5.32)
Quintile 4	3.14**	(1.72, 5.75)	2.86**	(1.53, 5.33)			2.89**	(1.56, 5.33)
Quintile 5	3.02**	(1.56, 5.84)	3.69**	(1.74, 7.81)			3.17**	(1.46, 6.86)
Social assistance								
No	1		1				1	
Yes	5.66**	(4.03, 7.97)	2.84**	(1.88, 4.30)			2.35**	(1.59, 3.49)
Residence								
Urban	1		1				1	
Rural	0.78	(0.58, 1.05)	0.59**	(0.42, 0.82)			0.65*	(0.46, 0.93)
Smoking								
No	1				1		1	
Yes	0.49**	(0.34, 0.71)			0.75	(0.50, 1.12)	0.61*	(0.38, 0.96)
Obesity								
Not obese	1				1		1	
Obese	4.80**	(3.53, 6.52)			4.28**	(3.12, 5.88)	2.33**	(1.60, 3.39)
Depression	1.09**	(1.05, 1.13)			1.07**	(1.03, 1.11)	1.07**	(1.02, 1.11)
Health facility visits								
No	1				1		1	
Yes	8.05**	(5.98, 10.84)			6.91**	(5.07, 9.43)	5.14**	(3.75, 7.05)
Civic participation								
No	1				1		1	
Yes	1.28	(0.92, 1.79)			1.10	(0.78, 1.56)	1.03	(0.77, 1.38)

**p<0.001; *p<0.05; *OR* Odds ratio, *CI* Confidence interval.

In step 2 (structural factors), the variables significantly associated with 1 chronic disease were age (1.18; 1.14-1.21), gender (1.53; 1.30-1.80), employment (0.70; 0.58-0.86), social assistance (1.48; 1.22-1.80) and residence (0.65; 0.52-0.81). Employment and residence were negatively associated with 1 chronic disease. This pattern was similar to that observed for multimorbidity, with age (1.44; 1.32-1.57), gender (1.95; 1.39-2.73), employment (0.65; 0.42-0.99), social assistance (2.84; 1.88-4.30) and residence (0.59; 0.42-0.82) being the significant factors. Additionally, household income was strongly associated with multimorbidity, a positive relationship implying that persons from high income households were more likely to have 2 or more chronic diseases.

In step 3 (intermediary factors), smoking, which was a significant univariate factor for multimorbidity, was not associated with neither 1 nor 2 or more chronic diseases. Civic participation was also consistently not associated with morbidity. The variables associated with both 1 chronic disease and multimorbidity were obesity, depression and health facility visits.

When all the variables were included in the analysis in step 4 (full model), gender, race, education, income, smoking and civic participation were not associated with 1 chronic disease. While employment was strongly associated with 1 chronic disease (0.72; 0.58-0.90), it was not significant for multimorbidity. Income was also strongly associated with multimorbidity, but not 1 chronic disease. Whereas smoking was not a factor with 1 chronic disease, it was negatively associated with multimorbidity (0.61; 0.38-0.96). Other variables significantly associated with multimorbidity were age (1.38; 1.25-1.51), social assistance (2.35; 1.59-3.49), residence (0.65; 0.46-0.93), obesity (2.33; 1.60-3.39), depression (1.07; 1.02-1.11) and health facility visits (5.14; 3.75-7.05).

## Discussion

This paper used data from a national household survey to examine the prevalence of multimorbidity and its associated socio-economic and demographic determinants. The results suggest that several factors including age, gender, education, income, employment, obesity, and depression are associated with both single and multimorbidity. Income and smoking, may not be associated with having a single chronic disease, but are strongly associated with multimorbidity. The magnitudes of the coefficients seem to also be larger for multimorbidity compared to single chronic disease, signifying a higher likelihood of an association. To our knowledge, this is the first study in South Africa, which indicates these associations at a population level. The findings will be important to healthcare planning and decision making and may have an impact on the management of non-communicable diseases.

### Study strengths and limitations

A major strength of this study was the use of data from a large nationally representative sample. This made it possible to examine relationships between multimorbidity and various social determinants of health. However, the cross-sectional nature of the study limits the ability to draw causal inferences.

The study could have underestimated the prevalence of multimorbidity, since only a few chronic diseases were included. Further, the chronic diseases assessed in this study were reported by the respondents and not measured independently by an expert. While it is possible for respondents to know of their afflictions, this cannot be taken with the certainty that would be given if the diseases were measured objectively. Since the diseases were reported and not measured, it is difficult to judge whether the diseases present in an individual were active or relevant. As a result, there was no differentiation of the weight of the various diseases within an individual. The measure of multimorbidity was thus a count that did not take into account the intensity or severity of disease. The study also misses out on the hidden chronic diseases, which may be unknown to patients, and discovered only by expert diagnosis.

In this paper, we define multimorbidity as the coexistence of multiple diseases. This definition is made from a limited, medicalised perspective, which may not take into account the patients’ understanding of their problems. A more wholistic definition is required to capture a representative view of disease. This, however, was not possible in this analysis given the way that data were collected in the SA-NIDS.

### Comparison with other studies

Estimates of multimorbidity prevalence vary from country to country due to settings and definitions of multimorbidity. However, a recent systematic review of studies in developed countries by Fortin et al., indicated a prevalence rate ranging from 3.5% to 98.5% in primary care setting and 13.1% to 71.8% nationally [34]. In our study, the prevalence of multimorbidity was 4%, with over 70% of adults with multimorbidity being females. Other studies also show that multimorbidity may be more common in females than males [35]. This finding warrants further investigations into gender differentials and the effects of gender-based inequities in the health sector in sub-Saharan African countries. Health inequalities have been shown to exist in the South African population [23], as well as the gender disparities in health services utilisation and the associated health outcomes [36].

The study found a positive association between household income and multimorbidity (but not having a single chronic disease). This association remained strong, even after adjusting for other variables. Other studies have shown this relationship between household income and multimorbidity [27], but some show that the reverse is also true [37]. In many low and middle income countries, non-communicable diseases are shown to be present in individuals from affluent communities and well-to-do households, which are more likely to adopt Western lifestyles and diets [19].

Chronic diseases, both single and multimorbidity, were less common among educated and employed persons. Empirical population-based studies mostly from high income countries indicate that better educated individuals have less risk of chronic diseases such as cardiovascular diseases, as well as multimorbidity [38,39]. Improved education could reflect a better use of information that improves access to healthcare and also indirectly reduces behavioural risk factors [39]. Informal education, which happens at the family and community level, through interactions with others, is also a source of information that can have an impact on one’s health status. Indeed, features of social organization, such as interpersonal trust, reciprocity norms, and engagements with community and neighbourhood, known as social capital, have been shown to be beneficial for health [40]. In our study, however, social capital was consistently not associated with health. Even though social capital (measured by network membership) has been found to play a significant role in an individual’s well-being [40], its relationship with health outcomes varies from being protective in nature [41] to lack of an association [33] and even sometimes destructive [42]. Though not statistically significant, the paradox can be observed between social networks and multimorbidity where civic participation increased the odds of multimorbidity. However, the inconsistency in the nature of association between social capital and health outcomes observed across literature may be due to variations in definitions and measurements [43]. It should also be noted that the measure of social capital used in this study did not include family ties, which have been shown to be important to managing chronic diseases [20]. Future research should test for the effect of family ties, as this could have an impact on the occurrence of multimorbidity.

As expected, obesity was positively associated with multimorbidity. This reiterates the fact that obesity is an underlying risk factor for a number of chronic diseases and multimorbidity. Obesity should therefore be a serious public health concern because of its negative effects on the health system [10] especially in Africa [12]. We also found that healthcare utilisation was positively associated with multimorbidity. This might be the case, because people who consult more often might have more conditions diagnosed [44].

### Policy implications

The study findings give important insight into socio-economic and demographic factors associated with multimorbidity in South Africa. With the on-going health sector reforms in the country, there is need for policy makers to consider the implications of multimorbidity on the health system and its impact on healthcare resources, particularly in the face of increasing obesity and a high burden of HIV/AIDS. This study provides useful information that can aid decision making and resource allocation for improved health outcomes. For instance, the association between education, employment and multimorbidity may imply that people with better individual economic capability could have more resources to take care of themselves and thereby reduce the risk of multiple chronic diseases regardless of their age. We have shown that obesity and depression are associated with chronic disease and multimorbidity. There is need to put in place measures that will encourage healthy lifestyle and living in the general population. Mental illness, which is often a neglected disease, should be brought to the fore, in order to address its negative impact.

## Conclusion

Given the paucity of empirical research on the social determinants of health and multimorbidity in Africa, this study adds important evidence to the literature on this topical issue. Due to the fact that research, medicine and health care has frequently focused on single disease [45,46], not much is known about the correlates of multimorbidity in many low and middle income countries [47]. It is important though, to adequately document this evidence, because it could have implications on the way resources are distributed to produce better health outcomes. In our study, we show that though similar, the correlates of chronic disease may differ from those of multimorbidity. For example, income appears to be more relevant to multimorbidity, and addressing welfare issues may be crucial to reducing multimorbidity. There could also be a difference in the way several other factors relate with single disease and multimorbidity, based on the strength and magnitude of the associations. This should be investigated in future research on the factors affecting multimorbidity.

## Competing interest

Both authors declare that they have no conflict of interest.

## Authors’ contributions

Both authors contributed to the conceptualization and development of the theoretical ideas. OA and LC performed the analysis, wrote, read and approved the final manuscript.
